# Associations Between Polymorphisms in Genes Related to Oxidative Stress and DNA Repair, Interactions With Serum Antioxidants, and Prostate Cancer Risk: Results From the Prostate Cancer Prevention Trial

**DOI:** 10.3389/fonc.2021.808715

**Published:** 2022-01-14

**Authors:** Zhihong Gong, Mary E. Platek, Cathee Till, Phyllis J. Goodman, Catherine M. Tangen, Elizabeth A. Platz, Marian L. Neuhouser, Ian M. Thompson, Regina M. Santella, Christine B. Ambrosone

**Affiliations:** ^1^ Department of Cancer Prevention & Control, Roswell Park Comprehensive Cancer Center, Buffalo, NY, United States; ^2^ Division of Public Health Sciences, Fred Hutchinson Cancer Research Center, Seattle, WA, United States; ^3^ Department of Epidemiology, Johns Hopkins Bloomberg School of Public Health, Baltimore, MD, United States; ^4^ Sidney Kimmel Comprehensive Cancer Center, Johns Hopkins University, Baltimore, MD, United States; ^5^ Department of Urology, University of Texas Health Science Center, San Antonio, TX, United States; ^6^ Department of Environmental Health Sciences and Herbert Irving Comprehensive Cancer Center, Columbia University, New York, NY, United States

**Keywords:** prostate cancer, genetic polymorphisms, DNA repair, oxidative stress, serum antioxidant

## Abstract

Study of polymorphisms in genes related to the generation and removal of oxidative stress and repair of oxidative DNA damage will lead to new insights into the genetic basis of prostate cancer. In the Prostate Cancer Prevention Trial (PCPT), a double-blind, randomized controlled trial testing finasteride versus placebo for prostate cancer prevention, we intend to investigate the role of oxidative stress/DNA repair mechanisms in prostate cancer etiology and whether these polymorphisms modify prostate cancer risk by interacting with antioxidant status in both placebo and finasteride arms. We evaluated associations of selected candidate polymorphisms in genes in these pathways, and interactions with pre-diagnostic serum antioxidants, and the risk of prostate cancer among 1,598 cases and 1,706 frequency-matched controls enrolled in the PCPT. Odds ratios (ORs) and 95% confidence intervals (CIs) were estimated using multivariable-adjusted logistic regression models. While there were no statistically significant associations observed in the placebo arm, several SNPs were associated with prostate cancer in the finasteride arm. Specifically, *APEX1*-rs1760944 was associated with increased risk of total prostate cancer (per minor allele: p-trend=0.04). *OGG1*-rs1052133 was positively (CG/GG vs. CC: OR=1.32, 95% CI: 1.01-1.73) and *NOS3*-rs1799983 was inversely (per minor allele: p-trend=0.04) associated with risk of low-grade prostate cancer. *LIG3*-rs1052536 and *XRCC1*-rs25489 were suggestively associated with reduced risk of high-grade prostate cancer (per minor allele: both p-trend=0.04). In the placebo arm, significant associations were observed among men with higher serum lycopene for *APEX1*-rs1760944 and *NQO1*-rs1800566, or higher serum β-cryptoxanthin for *ERCC4*-rs1800067. In the finasteride arm, stronger associations were observed among men with lower serum lycopene for *NOS3*-rs1799983, higher serum α-carotene, β-carotene, and β-cryptoxanthin for *LIG3*-rs1052536, or lower serum retinol for *SOD2*-rs1799725. These results suggest that germline variations in oxidative stress and DNA repair pathways may contribute to prostate carcinogenesis and that these associations may differ by intraprostatic sex steroid hormone status and be further modified by antioxidant status. Findings provide insights into the complex role of gene, gene-antioxidant and -finasteride interactions in prostate cancer etiology, and thus may lead to the development of preventative strategies.

## Introduction

Prostate cancer remains the most commonly diagnosed cancer and the second leading cause of cancer death in men among the majority of countries, including the United States (U.S.) (1, 2). It is estimated that during 2021, 248,530 new cases will be diagnosed in the U.S. and 34,130 men will die from the disease (3). Incidence is expected to rise along with the aging male population. To date, the etiology of prostate cancer remains largely unclear, however, oxidative stress has been implicated in prostate carcinogenesis (4).

Evidence from epidemiological, clinical and experimental studies suggest prostate cancer cells are exposed to increased levels of oxidative stress (5, 6). Many of the known and suspected risk factors for prostate cancer such as age are associated with higher levels of reactive oxygen species (ROS) and/or decreased antioxidant capabilities (7, 8). Oxidative stress, an imbalance between ROS and antioxidant capacity, leads to damaged DNA and mutations. The damage caused by oxidative stress can be further exacerbated by diminished antioxidant defense mechanisms (9). Various DNA repair systems can remedy this situation before cell replication and mutation fixation, but inherent variation can lead to diminished DNA repair capacity (10, 11). Additionally, oxidative stress and DNA repair capacity might interact with serum levels of antioxidants, resulting in an increased or reduced antioxidant/DNA repair capacity, which, in turn, influence prostate cancer risk.

Using data and biospecimens from the Prostate Cancer Prevention Trial (PCPT) (12, 13), a double-blind randomized controlled trial testing finasteride versus placebo for prostate cancer prevention, this study investigated candidate polymorphisms in multiple key genes involved in oxidative stress and DNA repair pathways with risk of prostate cancer. With measurement of serum antioxidant concentrations available among PCPT participants (14, 15), we further examined whether these associations differed by circulating antioxidant levels including carotenoids and retinol. PCPT included biopsy-determined presence or absence of prostate cancer, uniform pathologic assessment of tumor stage and grade, and measurement of serum antioxidants, allowed investigation of these associations with total, low-, and high-grade prostate cancer in both placebo and finasteride arms, and additional analysis to examine whether associations were modified by levels of serum antioxidants. Findings from this study could provide further insight into the genetics of oxidative stress and DNA repair mechanisms in prostate carcinogenesis among men both in the placebo arm and those who received finasteride for prostate cancer prevention.

## Materials and Methods

### Study Design and Study Population

We conducted a case-control study nested in the PCPT, a randomized, placebo-controlled trial testing whether the 5α-reductase inhibitor finasteride could reduce the 7-year period prevalence of prostate cancer (13). Characterizing and clinical data and stored blood specimens were collected during the trial. Details of the PCPT study design and characteristics of the study population have been described previously (12, 13, 16). In brief, men aged ≥ 55 years (n=18,882) with normal digital rectal examination (DRE), normal prostate-specific antigen (PSA) level (≤ 3 ng/mL) and without history of prostate cancer or severe benign prostatic hyperplasia related symptoms or other clinically significant and related conditions were randomized to receive either finasteride (5 mg/day) or placebo. All participants underwent DRE and PSA testing annually and those with abnormal findings were recommended for biopsy. At the end of the 7-year study period, all participants who were not previously diagnosed with prostate cancer were recommended to undergo an end-of-study biopsy. All biopsies were collected under transrectal ultrasonographic guidance and involved a minimum of six specimens (cores). All biopsies were reviewed to confirm the diagnosis of adenocarcinoma by both the pathologist at the local study site and at a central pathology laboratory. Discordant pathology interpretations were arbitrated by a referee pathologist, and concordance was achieved in all cases. Clinical stage was assigned locally. Tumors were graded centrally using the Gleason scoring system. Tumors with Gleason sum <7 were classified as low-grade and those with scores ≥7 were classified as high-grade. In this nested case-control study within PCPT, all eligible cases were included, and controls were selected from men with a negative biopsy at the end of study, and frequency matched to cases on age, treatment arm, and first-degree family history of prostate cancer, and were oversampled on race to include all eligible non-whites. A total of 1,598 cases and 1,706 controls had adequate DNA for genotyping and were included in the final analysis. Participants excluded due to inadequate DNA were comparable in age, body mass index (BMI), race, family history and treatment to those included in the analysis.

The Institutional Review Boards of all participating SWOG institutions approved this study and all participants signed written informed consent.

### Data and Specimen Collection

Self-administered questionnaires were used following recruitment and consent to collect information concerning age, race, education, physical activity, smoking, alcohol consumption and family history of prostate cancer. At the baseline clinic visit, approximately 3 months prior to randomization, height and weight were measured and non-fasting blood was collected. At each annual clinic visit, weight was measured, and non-fasting blood was collected either until prostate cancer diagnosis or the end of the study. Detailed procedures for blood collection, processing, and storage have been previously described (17).

### Laboratory Assessments

For the nested case-control study, the Qiagen M48 robot (Valencia, CA) was used to extract DNA from white blood cells at NCI Frederick and subsequently shipped to Roswell Park Comprehensive Cancer Center Genomics Core for genotyping by polymerase chain reaction (PCR) amplification followed by Capillary Electrophoresis fragment analysis. All primers were acquired from Applied Biosystems (Foster City, CA). All PCR amplifications were performed in 96-well plates using a Bio-Rad DNA Engine thermal cycler (Hercules, CA). We focused on two pathways and concentrated efforts on single nucleotide polymorphisms (SNPs) for which prior phenotypic or epidemiological data suggest an association with risk for prostate or other cancers. Specifically, based on information obtained in the literature at the time, we selected a panel of 21 potentially functional SNPs in genes related to oxidative stress and DNA repair pathways. After removing SNPs with call rate <95% (4 SNPs) or violation of Hardy Weinberg Equilibrium (1 SNP), a total of 16 SNPs in five genes of the oxidative stress pathway (*NOS3, NQO1, GSTP1, GSTA1, SOD2*) and in seven genes related to DNA repair pathway (*APEX1, OGG1, XRCC1, XRCC3, LIG3, XPD, ERCC4*) were included in the analysis. DNA was extracted from serum (for participants without stored buffy coat) and white blood cells (WBC); 8 SNPs were genotyped in WBC DNA, and the other 8 SNPs were genotyped in both WBC and serum DNA; comparison of results from analyses for combined samples and for samples separated by source showed no difference. Therefore, results reported are from analysis of combined genotyping data from WBC and serum DNA.

Pre-diagnostic total serum concentrations, including all *tans* and *cis* forms, of carotenoids (lycopene, α- and β-carotene, and β-cryptoxanthin) and retinol were measured by the high-performance liquid chromatography, among men in this nested case-control study, and their associations with prostate cancer risk were reported previously (14, 15). The weighted average coefficients of variation for pooled quality control samples were 13.5% for lycopene, 13% for α-carotene, 13% for β-carotene, 15% for β-cryptoxanthin, and 8% for retinol.

### Statistical Analysis

All analyses were performed separately in the placebo and finasteride arm given the *a priori* hypotheses that finasteride use may affect oxidative stress and further modify SNP-prostate cancer associations. Differences in demographic and participant characteristics between prostate cancer cases and controls were tested using standard chi square tests for categorical variables in the placebo and finasteride arms, respectively. Median concentrations and the interquartile ranges for the blood analytes were compared between cases and controls using the Wilcoxon rank-sum test. Multivariable logistic regression was used to estimate odds ratios (ORs) and 95% confidence intervals (CIs) for the risk of prostate cancer overall. When dependent variable has three categories, polytomous logistic regression was used to estimate risk for low-grade and high-grade prostate cancer, compared with controls. All models were adjusted for baseline age, race, education, BMI, family history of prostate cancer, diabetes, smoking, physical activity, and alcohol consumption. Participants with the most common homozygous genotype among controls were treated as the referent group. Genotypic (co-dominant) models were assumed for SNP effects. Based on the risk estimates, heterozygotes were combined with either homozygous rare or homozygous common genotypes to explore dominant and recessive models, respectively. Additive genotype coding on the number of rare alleles was analyzed as an ordinal variable in tests for linear trend. To test for potential effect modification, genotype was used as an ordinal variable and antioxidants levels were dichotomized into low and high categories based on the median concentration in the control population. Interaction was tested by including an interaction term genotype*antioxidant in multivariable logistic models. The significance of the interaction term (p-interaction) was tested using a likelihood ratio test, which compares the log likelihoods of the two models (i.e., with and without the interaction term) and tests whether this difference is statistically significant. All analyses were two sided and statistical significance was defined as p<0.05; analyses were performed using SAS 9.4 (SAS institute, Cary, NC).

## Results

### Participant Characteristics

Descriptive characteristics were compared between the prostate cancer cases and controls in the placebo and finasteride arms (**Table 1**). Study participants were primarily White (86% of the total cohort). Compared with controls, cases were less likely to be diagnosed with diabetes in the placebo arm, and more likely to be well-educated in the finasteride arm. Other factors were comparable between cases and controls in both arms. In the placebo arm, serum antioxidant concentrations showed significant differences for α-carotene, β-carotene and retinol between cases and controls, although actual differences were minimal. As shown, cases had a statistically significant higher median level of serum α-carotene (0.05 vs. 0.04 μg/mL), β-carotene (0.24 vs. 0.22 μg/mL), and retinol (0.69 vs. 0.67 μg/mL) than controls. Because results were similar for the entire cohort and for White only, we report results for the entire cohort.

**Table 1:** Baseline characteristics of participants^1^ in the Prostate Cancer Prevention Trial (PCPT).

Characteristic	Placebo			Finasteride		
Frequency, n (%)	Controls (n = 986)	Cases (n = 933)	p- value^3^	Controls (n = 720)	Cases (n = 665)	p- value^3^
Age (years)			0.98			0.96
< 60	265 (26.9)	258 (27.6)		190 (26.4)	178 (26.7)	
60-<65	325 (33.0	304 (32.6)		233 (32.4)	207 (31.1)	
65-<70	236 (23.9)	218 (23.4)		173 (24.0)	165 (24.8)	
70+	160 (16.2)	153 (16.4)		124 (17.2)	115 (17.3)	
Family history of prostate cancer	203 (20.6)	188 (20.2)	0.81	159 (22.1)	144 (21.6)	0.85
White	834 (84.6)	869 (93.1)		526 (73.1)	618 (92.9)	
Diabetes	72 (7.3)	36 (3.9)	0.001	54 (7.5)	35 (5.3)	0.09
Education			0.24			0.01
High school	176 (17.8)	152 (16.3)		151 (21.0)	115 (17.3)	
College	293 (29.7)	256 (27.4)		219 (30.4)	174 (26.2)	
Advanced degree	517 (52.4)	525 (56.3)		350 (48.6)	376 (56.5)	
Body mass index (BMI)			0.02			0.96
<25	236 (23.9)	273 (29.3)		193 (26.8)	174 (26.2)	
25-29	538 (54.6)	487 (52.2)		366 (50.8)	340 (51.1)	
30+	212 (21.5)	173 (18.5)		161 (22.4)	151 (22.7)	
Physical activity			0.14			0.59
Sedentary	158 (16.0)	147 (15.8)		135 (18.8)	116 (17.4)	
Light	416 (42.2)	375 (40.2)		290 (40.3)	289 (43.5)	
Moderate	301 (30.5)	326 (34.9)		228 (31.7)	207 (31.1)	
Activate	111 (11.3)	85 (9.1)		67 (9.3)	53 (8.0)	
Alcohol drinking (g/day), quartiles			0.86			0.84
<0.20	252 (25.6)	233 (25.0)		180 (25.0)	160 (24.1)	
0.20-<3.13	248 (25.2)	225 (24.1)		171 (23.4)	148 (22.3)	
3.13-<12.5	241 (24.4)	228 (24.4)		190 (26.4)	185 (27.8)	
≥12.5	245 (24.8)	247 (26.5)		179 (24.9)	172 (25.9)	
Smoking Status			0.58			0.63
Never smoker	339 (34.4)	339 (36.3)		245 (34.0)	221 (33.2)	
Former smoker	572 (58.0)	531 (56.9)		419 (58.2)	400 (60.2)	
Current smoker	75 (7.6)	63 (6.8)		56 (7.8)	44 (6.6)	
Serum antioxidants, median (IQR)^2^						
Lycopene, ug/mL	0.36 (0.26-0.46)	0.37 (0.28-0.48)	0.22	0.35 (0.26-0.47)	0.35 (0.26-0.47)	0.93
α-carotene, ug/mL	0.04 (0.02-0.07)	0.05 (0.03-0.07)	0.002	0.04 (0.03-0.07)	0.05 (0.03-0.07)	0.31
β-carotene, ug/mL	0.23 (0.14-0.36)	0.24 (0.10-0.38)	0.003	0.23 (0.14-0.38)	0.24 (0.16-0.37)	0.21
β-cryptoxanthin, ug/mL	0.08 (0.06-0.12)	0.09 (0.06-0.12)	0.64	0.09 (0.06-0.12)	0.09 (0.06-0.12)	0.84
Retinol, ug/mL	0.67 (0.58-0.77)	0.69 (0.60-0.79)	0.01	0.68 (0.58-0.78)	0.68 (0.59-0.80)	0.32

^1^Nested case-control selection: frequency matched on age, first-degree family history of prostate cancer and treatment arm, and oversampled on race to include all non-white controls.

^2^IQR, interquartile range; µg, micrograms.

^3^Based on Wilcoxon rank-sum test for continuous variables and Chi-square test for categorical variables.

### Associations of SNPs With Prostate Cancer Risk in the Placebo and Finasteride Arms

Associations between each SNP and risk of prostate cancer in the placebo and finasteride arms are shown separately in **Supplemental Tables S1, S2**. While there were no statistically significant associations observed in the placebo arm, several SNPs were associated with prostate cancer in the finasteride arm; ORs (95% CIs) with significant associations are shown in **Table 2**. *APEX1*-rs1760944 was associated with increased risk of total prostate cancer (per minor allele: p-trend=0.04). *OGG1*-rs1052133 and *NOS3*-rs1799983 were associated with risk of low-grade prostate cancer. Specifically, an increased risk was observed for rs1052133 (CG/GG vs. CC: OR=1.32, 95% CI: 1.01-1.73) in the dominant model, and a reduced risk was observed for rs1799983 (per minor allele: p-trend=0.04). Additionally, there were suggestions that *LIG3*-rs1052536 and *XRCC1*-rs25489 were associated with reduced risk of high-grade prostate cancer (both per minor allele: p-trend=0.04).

**Table 2:** Significant associations between polymorphisms in oxidative stress and DNA repair genes and prostate cancer risk in PCPT finasteride arm.

Gene	SNP	Genotype	All prostate cancer			Low-grade prostate cancer			High-grade prostate cancer		
			Ca/Co^a^	OR (95%CI)^b,c^	p^d,e^	Ca^a^	OR (95%CI)^b,c^	p^d,e^	Ca^a^	OR (95%CI)^b,c^	p^d,e^
*APEX1*	rs1760944	GG	219/279	1.00	0.04	139	1.00	0.13	75	1.00	0.16
		TG	281/308	1.16 (0.91-1.49)		171	1.11 (0.84-1.48)		102	1.23 (0.87-1.74)	
		TT	111/102	1.41 (1.01-1.98)		67	1.35 (0.84-2.16)		36	1.35 (0.84-2.16)	
*OGG1*	rs1052133	CC	354/423	1.00	0.09	210	1.00	0.06	129	1.00	0.44
		CG	227/229	1.23 (0.96-1.57)		145	1.33 (1.01-1.76)		77	1.12 (0.80-1.57)	
		GG	33/37	1.27 (0.75-2.15)		19	1.27 (0.69-2.34)		12	1.20 (0.59-2.44)	
		CG/GG	260/266	1.23 (0.98-1.56)	0.08	164	1.32 (1.01-1.73)	0.04	89	1.13 (0.82-1.56)	0.45
*NOS3*	rs1799983	GG	289/344	1.00	0.25	189	1.00	0.04	93	1.00	0.67
		GT	273/264	1.08 (0.84-1.38)		154	0.93 (0.70-1.24)		106	1.31 (0.94-1.83)	
		TT	57/81	0.68 (0.46-1.01)		31	0.57 (0.36-0.90)		24	0.90 (0.53-1.53)	
*LIG3*	rs1052536	CC	205/251	1.00	0.24	121	1.00	0.60	80	1.00	0.04
		CT	310/310	1.04 (0.80-1.34)		185	1.06 (0.78-1.43)		114	0.97 (0.68-1.37)	
		TT	109/133	0.79 (0.57-1.10)		72	0.88 (0.60-1.29)		30	0.56 (0.35-0.91)	
*XRCC1*	rs25489	GG	403/495	1.00	0.62	248	1.00	0.50	142	1.00	0.04
		AG	32/48	0.84 (0.52-1.37)		26	1.12 (0.66-1.90)		5	0.37 (0.14-0.97)	
		AA	2/2	1.40 (0.18-10.9)		2	2.18 (0.27-17.4)		0	n/a	
		AG/AA	34/50	0.86 (0.53-1.39)	0.54	28	1.16 (0.69-1.94)	0.58	5	0.36 (0.14-0.93)	0.04

a Ca/Co, Cases and Controls.

b OR, odds ratio; 95%CI, 95% confidence interval.

c Adjusted for baseline age, race, education, body mass index, family history of prostate cancer, diabetes status, smoking status, physical activity, and alcohol consumption.

d P-trend for genetic dose response determined by coding genotypes as having 0, 1, or 2 variant allele, which was subsequently analyzed as an ordinal variable.

e P for heterogeneity from dominant or recessive models.

### Effect Modification by Pre-Diagnostic Serum Antioxidant Concentrations

We next examined whether any SNP-prostate cancer risk associations were modified by serum concentrations of carotenoids and retinol within each treatment arm. As shown in **Tables 3** and **4**, in both the placebo and finasteride arms, there were indications that associations for several SNPs were modified by serum levels of lycopene and other antioxidants (p-interaction<0.05). In the placebo arm, although there was no significant main effect, *APEX1*-rs1760944 was associated with increased risk among men in the higher-level lycopene group, whereas *NQO1*-rs1800566 and *ERCC4*-rs1800067 were associated with decreased risk among men in the higher-level of lycopene and β-cryptoxanthin group, respectively (**Table 3**). In the finasteride arm, significant associations were observed for SNPs in *APEX1*, *NOS3*, *LIG3*, and *SOD2* among men in the lower- or higher-level group of serum antioxidants (**Table 4**). For example, a reduced risk was observed for *NOS3*-rs1799983 among men with lower levels of lycopene, and a somewhat increased risk among those with higher levels (p-interaction=0.03). We observed *LIG3*-rs1052536 associated with decreased risk among men with higher levels of all three provitamin A carotenoids (α-carotene, β-carotene, and β-cryptoxanthin). In addition, *SOD2*-rs1799725 was associated with increased risk among men with lower levels of retinol, but not among men with higher levels. Interestingly, consistent with results in the placebo arm, there was a suggestion that associations of *APEX1*-rs1760944 was modified by levels of antioxidants in the finasteride arm, although interactions were not statistically significant; significant increased risk was observed among men with higher levels of lycopene (p-trend=0.02), lower levels of α-carotene (p-trend=0.02) and retinol (p-trend=0.006), with a p-interaction of 0.32, 0.25, and 0.07, respectively.

**Table 3:** Associations of SNPs with prostate cancer risk stratified by serum antioxidants in PCPT placebo arm.

Gene	SNP	Genotype	Ca/Co^a^	OR (95%CI)^b,c^	p^d,e^	Ca/Co^a^	OR (95%CI)^b,c^	p^d,e^	P-inter^f^
**Lycopene**			**Low-level (<0.38 µg/mL)**			**High-level (≥0.38 µg/mL)**			
*APEX1*	rs1760944	GG	167/166	1.00	0.08	137/171	1.00	0.006	0.002
		TG	220/252	0.83 (0.62-1.11)		200/198	1.29 (0.94-1.76)		
		TT	64/87	0.72 (0.48-1.07)	0.12	81/60	1.80 (1.18-2.75)		
*NQO1*	rs1800566	CC	300/337	1.00	0.45	285/245	1.00	0.04	0.04
		CT	123/157	0.95 (0.71-1.27)		113/153	0.69 (0.50-0.94)		
		TT	26/19	1.65 (0.88-3.10)		21/28	0.78 (0.41-1.46)		
**β-cryptoxanthin**			**Low-level (<0.10 µg/mL)**			**High-level (≥0.10 µg/mL)**			
*ERCC4*	rs1800067	GG	325/417	1.00	0.66	237/224	1.00	0.01	0.02
		AG	47/52	1.10 (0.71-1.70)		27/52	0.48 (0.29-0.81)		
		AA	2/2	1.08 (0.14-8.22)		2/2	0.71 (0.10-5.23)		
		AG/AA	49/54	1.10 (0.72-1.69)	0.66	29/54	0.49 (0.30-0.82)	0.006	0.01

a Ca/Co, Cases and Controls.

b OR, odds ratio; 95%CI, 95% confidence interval.

c Adjusted for baseline age, race, education, body mass index, family history of prostate cancer, diabetes status, smoking status, physical activity, and alcohol consumption.

d P-trend for genetic dose response determined by coding genotypes as having 0, 1, or 2 variant allele, which was subsequently analyzed as an ordinal variable.

e P for heterogeneity from dominant or recessive models.

f P for interaction between genotype (ordinal variable)-serum level of antioxidants (low vs. high) using the likelihood ratio test.

**Table 4:** Associations of SNPs with prostate cancer risk stratified by serum antioxidants in PCPT finasteride arm.

Gene	SNP	Genotype	Ca/Co^a^	OR (95%CI)^b,c^	p^d,e^	Ca/Co^a^	OR (95%CI)^b,c^	p^d,e^	P*-*inter *^f^*
**Lycopene**			**Low-level (<0.38 µg/mL)**			**High-level (≥0.38 µg/mL)**			
*NOS3*	Rs1799983	GG	174/199	1.00	0.02	115/145	1.00	0.39	0.03
		GT	150/144	0.99 (0.71-1.37)		123/120	1.15 (0.79-1.68)		
		TT	28/53	0.43 (0.25-0.73)		29/28	1.24 (0.67-2.30)		
**α-carotene**			**Low-level (<0.06 µg/mL)**			**High-level (≥0.06 µg/mL)**			
*APEX1*	Rs1048945	GG	381/454	1.00	0.02	184/199	1.00	0.52	0.04
		CG	40/23	1.89 (1.09-3.27)		17/22	0.79 (0.39-1.62)		
		CC	1/0	n/a		0/0	n/a		
		CG/GG	41/23	1.94 (1.12-3.36)	0.02	17/22	0.79 (0.39-1.62)	0.52	0.05
*LIG3*	Rs1052536	CC	134/181	1.00	0.86	71/70	1.00	0.01	0.03
		CT	210/210	1.15 (0.84-1.58)		100/100	0.87 (0.54-1.40)		
		TT	77/84	1.00 (0.66-1.49)		32/49	0.44 (0.24-0.81)		
**β-carotene**			**Low-level (<0.30 µg/mL)**			**Low-level (≥0.30 µg/mL)**			
*LIG3*	Rs1052536	CC	127/172	1.00	0.59	78/79	1.00	0.006	0.01
		CT	205/210	1.16 (0.84-1.60)		105/100	0.84 (0.53-1.33)		
		TT	74/75	1.08 (0.71-1.64)		35/58	0.43 (0.24-0.77)		
**β-cryptoxanthin**			**Low-level (<0.10 µg/mL)**			**High-level (≥0.10 µg/mL)**			
*LIG3*	Rs1052536	CC	108/157	1.00	0.54	97/94	1.00	0.01	0.02
		CT	197/188	1.34 (0.96-1.87)		113/122	0.72 (0.47-1.10)		
		TT	71/81	1.08 (0.70-1.65)		38/52	0.49 (0.28-0.86)		
**Retinol**			**Low-level (<0.69 µg/mL)**			**High-level (≥0.69 µg/mL)**			
*SOD2*	Rs1799725	TT	40/74	1.00	0.005	51/57	1.00	0.47	0.02
		CT	121/141	1.56 (0.97-2.52)		119/159	0.64 (0.39-1.05)		
		CC	65/66	2.17 (1.26-3.75)		41/48	0.82 (0.44-1.53)		

a Ca/Co, Cases and Controls.

b OR, odds ratio; 95%CI, 95% confidence interval.

c Adjusted for baseline age, race, education, body mass index, family history of prostate cancer, diabetes status, smoking status, physical activity, and alcohol consumption

d P-trend for genetic dose response determined by coding genotypes as having 0, 1, or 2 variant allele, which was subsequently analyzed as an ordinal variable.

e P for heterogeneity from dominant or recessive models.

f P for interaction between genotype (ordinal variable) and serum level of antioxidants (low vs. high) using likelihood ratio test.

## Discussion

In this nested case-control study of 1,598 cases and 1,706 controls enrolled in the PCPT, we conducted an analysis of a panel of candidate genetic polymorphisms involved in the oxidative stress and DNA repair pathways with risk of prostate cancer in both placebo and finasteride arms. There were no significant associations observed in the placebo arm, however, in the finasteride arm, several SNPs in *APEX1*, *OGG1*, *NOS3*, *LIG3* and *XRCC1* were associated with risk of total, low- or high-grade prostate cancer. Our results also indicate that SNP-prostate cancer associations may be modified by levels of serval serum antioxidants and differ by treatment arm.

The *APEX1* encodes an enzyme involved in the DNA base excision repair pathway, which plays a central role in the cellular response to oxidative stress (18). Several studies have reported associations between *APEX1* SNPs, such as rs1760944 and rs1048945, and risk of several cancers including prostate (19–22). In this study of primarily White men, compared with the common G allele, the minor T allele of *APEX1*-rs1760944 was associated with increased risk of prostate cancer among men in the finasteride arm. In contrast, as shown in a meta-analysis, previous studies primarily conducted in Asian populations reported that the T allele is the common allele, with G allele associated with decreased risk of cancer including breast, lung and prostate (21). This polymorphism is located in the promoter region of *APEX1*, and there is evidence that the T allele is associated with altered promoter activity compared with the G allele (22). We previously reported there was no main effect of serum lycopene (14), whereas higher levels of serum retinol and α-carotene were associated with increased prostate cancer risk among men in the placebo arm (15). In this analysis, we observed that several SNP-prostate cancer associations were modified by serum levels of these antioxidants. We found that there was an increased prostate cancer risk among men who carry the rs1760944 variant T allele and have a higher-level of serum lycopene in both placebo and finasteride arm. An increased risk was also observed for the *APEX1-*rs1760944 and -rs1048945 among those with lower-levels of serum α-carotene and retinol. These results support a complex link between *APEX1* gene polymorphisms, serum level of different antioxidants, hormonal changes caused by finasteride use, and risk of prostate cancer, which warrants further investigations. Nevertheless, these findings suggest that *APEX1* variants may interact with antioxidant status to contribute to the susceptibility to prostate cancer.

Similar to *APEX1* SNPs, several SNPs in other DNA repair genes, specifically *OGG1*, *LIG3*, and *XRCC1*, were associated with prostate cancer among men taking finasteride. *OGG1* encodes a DNA glycosylase, which is critical for removal of the oxidatively damaged bases from DNA (23). We observed an increased risk for low-grade prostate cancer for rs1052133, which involves an amino acid substitution from serine to cysteine at codon 326 (Ser326Cys). This variant has been associated with reduced DNA repair capacity, and has previously linked with oxidation-related pathologies and a higher risk of cancer, including prostate (23–27). We also observed decreased risk for a SNP rs1052536 in *LIG3*, with a stronger association for high-grade prostate cancer. *LIG3* encodes a key ligase polypeptide that plays an essential role in the excision repair pathway. This SNP has been previously associated with the risk of young-onset lung cancer and an improved breast cancer survival (28, 29). Interestingly, we further found that the decreased risk associated with this SNP was limited to men with higher serum concentrations of each of the three provitamin A carotenoids (α-carotene, β-carotene, and β-cryptoxanthin). In addition, *XRCC1*-rs25489 was associated with a significant decreased risk for high-grade prostate cancer. Results from several studies that have examined this SNP with overall prostate cancer have been inconclusive (30, 31). These results provide additional evidence that these polymorphisms may modify prostate cancer risk by interacting with hormonal changes by finasteride use and serum antioxidants. It is intriguing that among men with finasteride use, certain SNPs were associated with reduced risk of prostate cancer, especially the aggressive high-grade cancer. There is some evidence that finasteride use causes hormonal alterations and hormones can modulate antioxidant defense system under various pathophysiological conditions (32, 33). It is possible that finasteride related hormonal changes can interact with higher serum levels of carotenoids to provide a stronger defense against oxidative stress, which may further lead to a stronger decreased risk of high-grade prostate cancer among men who carry specific genotypes of DNA repair genes. Future studies are needed to confirm these findings and underlying mechanisms.

We observed significant associations for two SNPs in genes involved in the oxidative stress pathways among men in the finasteride arm. The variant TT genotype of a common SNP on *NOS3*, rs1799983, was associated with a significant decreased risk for low-grade cancer. Results from several studies that have examined this variant in relation to prostate cancer have been inconsistent, and a meta-analysis suggested no significant association for prostate cancer overall (34). Interestingly, we found a reduced risk associated with this variant for low-grade cancer among men randomized to finasteride. Although the function of this variant in prostate carcinogenesis remains to be determined, rs1799983 located in exon 7 of *NOS3* gene, causing an amino acid substitution within the N-terminal oxygenase domain of NOS3 enzyme (34), may contribute to variability in oxidative stress and risk of prostate cancer by interacting with other genetic or hormonal factors in this subgroup population. We also observed that a SNP in *SOD2*, rs1799725, was associated with increased risk of prostate cancer, with a stronger association for men with low serum levels of retinol. *SOD2* encodes an antioxidant enzyme that converts reactive oxygen species to hydrogen peroxide and ultimately into water (35). The rs1799725 located in exon 2 of *SOD2*, also called rs4880, encodes for a thymine to cytosine (T to C allele) change, which causes an amino acid change from a valine to alanine at codon 16 (Val16Ala) (36). Consistent with our finding, a meta-analysis reported that this polymorphism was associated with increased risk for prostate cancer (37). Several previous studies also have reported that the SNP-prostate relationship may be modified by antioxidant status (38–40). These findings provide some evidence that the *SOD2*-rs1799725 may contribute to prostate cancer susceptibility, particularly in a low antioxidant environment.

Several limitations of the study warrant consideration. First, although we investigated a number of candidate SNPs in multiple key genes thought to be important in cancer risk in oxidative stress and DNA repair pathways, other potentially functional genetic variants were not included in the current study. Second, although this is a relatively large study, which allowed us to examine associations of these genetic variants with prostate cancer risk, our sample size was limited when analyses were stratified by low- and high- grade cancer, and by low- and high-levels of serum antioxidants, and may have been inadequate to detect a small effect size or interactions. Third, overall associations for main effects for each SNP are moderate, but results suggest that the interplay between genes involved in oxidative stress and DNA repair and prostate cancer risk may be modified by finasteride use and different levels of serum antioxidants. Fourth, we were unable to include other antioxidants other than carotenoids and retinol in the analysis. Finally, because of the hypothesis-driven nature of candidate SNP selection, we did not systematically correct for multiple testing in our analysis. There are also several strengths. First, PCPT was a large, placebo-controlled, randomized trial that all men in the study were determined by biopsy to have or not have cancer, which largely eliminates the possibility that controls may have had undiagnosed or undetected disease, minimizing misclassification bias. Second, all tumors were uniformly evaluated for Gleason score, which minimizes the large intra-observer variability in assigning clinical tumor grade. Lastly, we were able to examine associations by tumor grade and whether SNP-cancer associations were modified by antioxidant status in both placebo and finasteride arms, which may offer insights regarding the complex role of gene, gene-antioxidant and -finasteride interactions in prostate cancer risk, and thus may lead to the development of preventative strategies.

In conclusion, this study investigated the role of SNPs in oxidative stress and DNA repair genes as risk factors for prostate cancer overall and by tumor grade. Our study provides some evidence that genetic variants in these genes may contribute to risk of prostate cancer and may interact with antioxidant status and hormone changes caused by taking finasteride. Oxidative stress and DNA repair system are complex; thus, it might be a combined effect of both multiple genetic variants and their interactions with other factors such as host antioxidant and hormonal status that lead to cancer susceptibility. Additional functional evaluations are warranted to confirm these findings and explore the underlying molecular mechanisms.

## Data Availability Statement

The original contributions presented in the study are included in the article/**Supplementary Material**, further inquiries can be directed to the corresponding author.

## Ethics Statement

The studies involving human participants were reviewed and approved by The Institutional Review Boards of all participating SWOG institutions approved this study. The patients/participants provided their written informed consent to participate in this study.

## Author Contributions

ZG made substantial contributions to the data analysis, interpretation of the data, drafted the manuscript, and revised it critically for important intellectual content. MP, CT, PG, CMT, EP, MN, IT, RS, and CA were essential to study design, data collection and genotyping, interpretation of the data and manuscript writing. All authors approved this version to be published, have participated sufficiently in this work, and agree to be accountable for all aspects of the work in ensuring that questions related to the accuracy or integrity of any part of the work are appropriately investigated and resolved.

## Funding

This work was supported by grants from National Cancer Institute: K07 178293, U10 CA37429, R01 CA63164, UM1CA182883, P01 CA108964, and P30 CA016056.

## Conflict of Interest

The authors declare that the research was conducted in the absence of any commercial or financial relationships that could be construed as a potential conflict of interest.

## Publisher’s Note

All claims expressed in this article are solely those of the authors and do not necessarily represent those of their affiliated organizations, or those of the publisher, the editors and the reviewers. Any product that may be evaluated in this article, or claim that may be made by its manufacturer, is not guaranteed or endorsed by the publisher.
