# Supplementary material for: Associations Between Polymorphisms in Genes Related to Oxidative Stress and DNA Repair, Interactions With Serum Antioxidants, and Prostate Cancer Risk: Results From the Prostate Cancer Prevention Trial
Source: Front Oncol. 2022 Jan 14;11:808715. doi: 10.3389/fonc.2021.808715 (PMC8795906; doi:10.3389/fonc.2021.808715)
Supplement: Supplementary file 1 [file Table_1.docx]

Supplemental Table 1. Association between polymorphisms in oxidative stress and DNA repair genes and risk of prostate cancer in the

PCPT placebo arm

| **Gene** | **SNP** | **Genotype** | **All prostate cancer** | | | **Low-grade prostate cancer** | | | **High-grade prostate cancer** | | |
| --- | --- | --- | --- | --- | --- | --- | --- | --- | --- | --- | --- |
|  |  |  | **#Case/**  **Control** | **OR (95%CI)^b,c^** | ***P*^d,e^** | **#Cases** | **OR (95%CI)^b,c^** | ***P*^d,e^** | **#Cases** | **OR (95%CI)^b,c^** | ***P*^d,e^** |
| *APEX1* | Rs1760944 | CC | 304/337 | 1.00 | 0.49 | 220 | 1.00 | 0.24 | 74 | 1.00 | 0.36 |
|  |  | AC | 420/450 | 1.01 (0.82-1.25) |  | 328 | 1.08 (0.86-1.36) |  | 71 | 0.72 (0.50-1.04) |  |
|  |  | AA | 145/147 | 1.12 (0.84-1.49) |  | 112 | 1.20 (0.88-1.63) |  | 29 | 0.90 (0.56-1.46) |  |
|  | Rs1048945 | GG | 813/872 | 1.00 | 0.85 | 611 | 1.00 | 0.74 | 170 | 1.00 | 0.82 |
|  |  | CG | 71/78 | 0.95 (0.68-1.34) |  | 56 | 0.99 (0.69-1.43) |  | 12 | 0.78 (0.42-1.48) |  |
|  |  | CC | 3/0 | N/A |  | 2 | n/a |  | 1 | n/a |  |
|  |  | CG/CC | 74/78 | 0.99 (0.71-1.39) | 0.95 | 58 | 1.03 (0.72-1.48) | 0.88 | 13 | 0.86 (0.46-1.59) | 0.62 |
|  | Rs1130409 | TT | 259/274 | 1.00 | 0.28 | 193 | 1.00 | 0.26 | 54 | 1.00 | 0.94 |
|  |  | GT | 438/475 | 0.90 (0.72-1.13) |  | 337 | 0.92 (0.73-1.17) |  | 86 | 0.92 (0.63-1.34) |  |
|  |  | GG | 176/197 | 0.86 (0.66-1.14) |  | 130 | 0.84 (0.63-1.14) |  | 39 | 0.99 (0.63-1.58) |  |
| *XRCC1* | Rs25489 | GG | 573/666 | 1.00 | 0.49 | 443 | 1.00 | 0.66 | 111 | 1.00 | 0.25 |
|  |  | AG | 62/69 | 1.10 (0.76-1.60) |  | 47 | 1.09 (0.73-1.63) |  | 14 | 1.23 (0.66-2.31) |  |
|  |  | AA | 1/0 | N/A |  | 0 | N/A |  | 1 | N/A |  |
|  |  | AG/AA | 63/69 | 1.12 (0.77-1.62) | 0.55 | 47 | 1.09 (0.73-1.63) | 0.66 | 15 | 1.32 (0.72-2.43) | 0.37 |
|  | Rs25487 | GG | 369/406 | 1.00 | 0.94 | 271 | 1.00 | 0.99 | 79 | 1.00 | 0.88 |
|  |  | AG | 389/411 | 1.01 (0.82-1.24) |  | 303 | 1.06 (0.85-1.32) |  | 74 | 0.93 (0.65-1.32) |  |
|  |  | AA | 113/121 | 0.98 (0.72-1.32) |  | 85 | 0.97 (0.70-1.34) |  | 25 | 1.11 (0.67-1.85) |  |
|  | Rs1799782 | CC | 558/637 | 1.00 | 0.77 | 426 | 1.00 | 0.96 | 116 | 1.00 | 0.29 |
|  |  | CT | 84/109 | 0.92 (0.67-1.27) |  | 67 | 0.96 (0.69-1.35) |  | 13 | 0.72 (0.39-1.34) |  |
|  |  | CC | 2/1 | 2.77 (0.21-36.6) |  | 2 | 3.55 (0.26-47.8) |  | 0 | n/a |  |
|  |  | CT/CC | 86/110 | 0.94 (0.68-1.28) | 0.69 | 69 | 0.98 (0.70-1.38) | 0.93 | 13 | 0.72 (0.39-1.33) | 0.29 |
| *GSTA1* | Rs3957356 | CC | 222/255 | 1.00 | 0.96 | 166 | 1.00 | 0.88 | 50 | 1.00 | 0.52 |
|  |  | CT | 309/370 | 0.94 (0.74-1.19) |  | 241 | 0.96 (0.74-1.25) |  | 57 | 0.79 (0.52-1.20) |  |
|  |  | TT | 108/117 | 1.02 (0.74-1.41) |  | 84 | 1.05 (0.74-1.49) |  | 21 | 0.90 (0.51-1.59) |  |
| *GSTP1* | Rs947894 | AA | 267/310 | 1.00 | 0.65 | 213 | 1.00 | 0.99 | 48 | 1.00 | 0.52 |
|  |  | AG | 304/349 | 1.08 (0.85-1.36) |  | 225 | 0.99 (0.77-1.27) |  | 69 | 1.38 (0.92-2.08) |  |
|  |  | GG | 70/85 | 1.04 (0.72-1.51) |  | 54 | 1.02 (0.69-1.52) |  | 12 | 0.96 (0.48-1.91) |  |
| *XPD* | Rs28365048 | AA | 255/297 | 1.00 | 0.76 | 192 | 1.00 | 0.97 | 54 | 1.00 | 0.78 |
|  |  | AC | 295/347 | 0.96 (0.76-1.21) |  | 230 | 1.00 (0.77-1.28) |  | 56 | 0.86 (0.57-1.30) |  |
|  |  | CC | 82/88 | 0.97 (0.68-1.37) |  | 63 | 0.99 (0.68-1.45) |  | 18 | 1.00 (0.55-1.82) |  |
| *SOD2* | Rs1799725 | TT | 154/188 | 1.00 | 0.39 | 116 | 1.00 | 0.41 | 33 | 1.00 | 0.69 |
|  |  | CT | 319/376 | 1.02 (0.78-1.33) |  | 250 | 1.06 (0.79-1.41) |  | 59 | 0.88 (0.55-1.40) |  |
|  |  | CC | 161/176 | 1.15 (0.84-1.56) |  | 121 | 1.15 (0.82-1.61) |  | 35 | 1.11 (0.66-1.89) |  |
| *XRCC3* | Rs861539 | CC | 222/277 | 1.00 | 0.68 | 165 | 1.00 | 0.87 | 46 | 1.00 | 0.83 |
|  |  | CT | 311/339 | 1.07 (0.84-1.37) |  | 246 | 1.13 (0.87-1.47) |  | 59 | 0.99 (0.64-1.51) |  |
|  |  | TT | 83/107 | 0.88 (0.62-1.24) |  | 63 | 0.89 (0.61-1.30) |  | 18 | 0.93 (0.51-1.69) |  |
| *ERCC4* | Rs1800067 | GG | 562/641 | 1.00 | 0.12 | 433 | 1.00 | 0.15 | 113 | 1.00 | 0.32 |
|  |  | AG | 74/104 | 0.76 (0.54-1.05) |  | 55 | 0.73 (0.51-1.04) |  | 16 | 0.81 (0.46-1.44) |  |
|  |  | AA | 4/4 | 0.95 (0.23-3.86) |  | 4 | 1.27 (0.31-5.19) |  | 0 | n/a |  |
|  |  | AG/AA | 78/108 | 0.76 (0.55-1.05) | 0.10 | 59 | 0.75 (0.53-1.06) | 0.10 | 16 | 0.78 (0.44-1.38) | 0.38 |
| *LIG3* | Rs1052536 | CC | 264/301 | 1.00 | 0.77 | 201 | 1.00 | 0.78 | 56 | 1.00 | 0.37 |
|  |  | CT | 444/470 | 1.02 (0.82-1.27) |  | 339 | 0.99 (0.78-1.26) |  | 85 | 1.01 (0.69-1.49) |  |
|  |  | TT | 180/179 | 1.04 (0.79-1.37) |  | 131 | 0.96 (0.71-1.29) |  | 41 | 1.26 (0.79-2.00) |  |
| *OGG1* | Rs1052133 | CC | 526/587 | 1.00 | 0.42 | 400 | 1.00 | 0.52 | 111 | 1.00 | 0.71 |
|  |  | CG | 301/318 | 1.06 (0.87-1.30) |  | 222 | 1.03 (0.83-1.29) |  | 62 | 1.06 (0.75-1.50) |  |
|  |  | GG | 44/44 | 1.16 (0.74-1.82) |  | 34 | 1.19 (0.74-1.92) |  | 9 | 1.09 (0.51-2.33) |  |
| *NQO1* | Rs1800566 | CC | 585/582 | 1.00 | 0.30 | 455 | 1.00 | 0.12 | 107 | 1.00 | 0.45 |
|  |  | CT | 236/310 | 0.81 (0.65-0.99) |  | 165 | 0.73 (0.58-0.92) |  | 63 | 1.13 (0.80-1.60) |  |
|  |  | TT | 47/47 | 1.13 (0.73-1.75) |  | 36 | 1.11 (0.69-1.77) |  | 9 | 1.20 (0.56-2.55) |  |
| *NOS3* | Rs1799983 | GG | 388/443 | 1.00 | 0.24 | 291 | 1.00 | 0.28 | 89 | 1.00 | 0.90 |
|  |  | GT | 395/420 | 1.02 (0.84-1.25) |  | 296 | 1.00 (0.81-1.25) |  | 76 | 0.90 (0.64-1.27) |  |
|  |  | TT | 92/78 | 1.30 (0.93-1.82) |  | 71 | 1.31 (0.91-1.89) |  | 17 | 1.09 (0.61-1.96) |  |

^b^ OR, odds ratio; 95%CI, 95% confidence interval

^c^ Adjusted for baseline age, race, education, body mass index, family history of prostate cancer, diabetes status, smoking status, physical activity, and alcohol consumption.

^d^ *P-*trend for genetic dose response determined by coding genotypes as having 0, 1, or 2 minor allele, which was subsequently analyzed as an ordinal variable (additive model).

^e^ *P* for heterogeneity from dominant or recessive models.

Supplemental Table 2. Association between polymorphisms in oxidative stress and DNA repair genes and risk of prostate cancer in the

PCPT finasteride arm

| **Gene** | **SNP** | **Genotype** | **All prostate cancer** | | | **Low-grade prostate cancer** | | | **High-grade prostate cancer** | | |
| --- | --- | --- | --- | --- | --- | --- | --- | --- | --- | --- | --- |
|  |  |  | **#Case/**  **Control** | **OR (95%CI)^b,c^** | ***P*^d,e^** | **#Case/**  **Control** | **OR (95%CI)^b,c^** | ***P*^d,e^** | **#Case/**  **Control** | **OR (95%CI)^b,c^** | ***P*^d,e^** |
| *APEX1* | Rs1760944 | CC | 219/279 | 1.00 | **0.04** | 139 | 1.00 | 0.13 | 75 | 1.00 | 0.16 |
|  |  | AC | 281/308 | 1.16 (0.91-1.49) |  | 171 | 1.11 (0.84-1.48) |  | 102 | 1.23 (0.87-1.74) |  |
|  |  | AA | 111/102 | 1.41 (1.01-1.98) |  | 67 | 1.35 (0.84-2.16) |  | 36 | 1.35 (0.84-2.16) |  |
|  | Rs1048945 | GG | 565/653 | 1.00 | 0.12 | 339 | 1.00 | 0.14 | 208 | 1.00 | 0.54 |
|  |  | CG | 57/45 | 1.35 (0.89-2.06) |  | 37 | 1.43 (0.90-2.30) |  | 16 | 1.06 (0.58-1.94) |  |
|  |  | CC | 1/0 | n/a |  | 0 | n/a |  | 1 | n/a |  |
|  |  | CG/CC | 58/45 | 1.37 (0.90-2.09) | 0.14 | 37 | 1.43 (0.89-2.29) | 0.14 | 17 | 1.12 (0.62-2.04) | 0.70 |
|  | Rs1130409 | TT | 178/200 | 1.00 | 0.54 | 97 | 1.00 | 0.15 | 75 | 1.00 | 0.45 |
|  |  | GT | 273/357 | 0.79 (0.60-1.04) |  | 170 | 0.91 (0.66-1.25) |  | 93 | 0.64 (0.44-0.92) |  |
|  |  | GG | 157/129 | 1.14 (0.83-1.58) |  | 100 | 1.35 (0.93-1.95) |  | 52 | 0.90 (0.59-1.39) |  |
| *XRCC1* | Rs25489 | GG | 403/495 | 1.00 | 0.62 | 248 | 1.00 | 0.50 | 142 | 1.00 | **0.04** |
|  |  | AG | 32/48 | 0.84 (0.52-1.37) |  | 26 | 1.12 (0.66-1.90) |  | 5 | 0.37 (0.14-0.97) |  |
|  |  | AA | 2/2 | 1.40 (0.18-10.9) |  | 2 | 2.18 (0.27-17.4) |  | 0 | n/a |  |
|  |  | AG/AA | 34/50 | 0.86 (0.53-1.39) | 0.54 | 28 | 1.16 (0.69-1.94) | 0.58 | 5 | 0.36 (0.14-0.93) | 0.04 |
|  | Rs1799782 | CC | 395/487 | 1.00 | 0.48 | 241 | 1.00 | 0.10 | 139 | 1.00 | 0.41 |
|  |  | CT | 52/65 | 1.19 (0.78-1.80) |  | 38 | 1.49 (0.93-2.36) |  | 13 | 0.81 (0.42-1.55) |  |
|  |  | CC | 1/2 | 0.78 (0.07-9.28) |  | 1 | 1.35 (0.11-16.5) |  | 0 | n/a |  |
|  |  | CT/CC | 53/67 | 1.18 (0.78-1.78) | 0.44 | 39 | 1.48 (0.94-2.34) | 0.09 | 13 | 0.78 (0.41-1.50) | 0.46 |
|  | Rs25487 | GG | 267/326 | 1.00 | 0.96 | 166 | 1.00 | 0.75 | 92 | 1.00 | 0.53 |
|  |  | AG | 280/289 | 1.05 (0.82-1.34) |  | 170 | 1.02 (0.77-1.35) |  | 99 | 1.10 (0.78-1.54) |  |
|  |  | AA | 71/72 | 0.97 (0.66-1.42) |  | 40 | 0.89 (0.57-1.39) |  | 29 | 1.14 (0.68-1.89) |  |
| *GSTA1* | Rs3957356 | CC | 141/200 | 1.00 | 0.57 | 86 | 1.00 | 0.36 | 52 | 1.00 | 0.90 |
|  |  | CT | 234/263 | 1.24 (0.92-1.66) |  | 143 | 1.24 (0.88-1.74) |  | 81 | 1.18 (0.78-1.76) |  |
|  |  | TT | 72/84 | 1.09 (0.73-1.62) |  | 49 | 1.17 (0.74-1.84) |  | 20 | 0.87 (0.48-1.56) |  |
| *GSTP1* | Rs947894 | AA | 191/246 | 1.00 | 0.35 | 115 | 1.00 | 0.13 | 68 | 1.00 | 0.62 |
|  |  | AG | 199/239 | 1.12 (0.85-1.49) |  | 120 | 1.11 (0.80-1.54) |  | 74 | 1.17 (0.79-1.72) |  |
|  |  | GG | 55/65 | 1.18 (0.76-1.81) |  | 42 | 1.47 (0.91-2.36) |  | 10 | 0.62 (0.30-2.29) |  |
| *XPD* | Rs28365048 | AA | 167/217 | 1.00 | 0.92 | 108 | 1.00 | 0.58 | 53 | 1.00 | 0.32 |
|  |  | AC | 210/274 | 0.86 (0.65-1.14) |  | 130 | 0.82 (0.59-1.13) |  | 73 | 0.96 (0.64-1.44) |  |
|  |  | CC | 66/59 | 1.15 (0.75-1.76) |  | 36 | 0.98 (0.60-1.62) |  | 27 | 1.48 (0.84-2.60) |  |
| *SOD2* | Rs1799725 | TT | 92/131 | 1.00 | 0.07 | 57 | 1.00 | 0.13 | 35 | 1.00 | 0.33 |
|  |  | CT | 241/300 | 1.04 (0.75-1.45) |  | 154 | 1.09 (0.74-1.60) |  | 76 | 0.85 (0.54-1.35) |  |
|  |  | CC | 107/114 | 1.45 (0.97-2.15) |  | 64 | 1.43 (0.90-2.27) |  | 38 | 1.32 (0.77-2.27) |  |
| *XRCC3* | Rs861539 | CC | 176/246 | 1.00 | 0.34 | 109 | 1.00 | 0.35 | 62 | 1.00 | 0.73 |
|  |  | CT | 204/250 | 1.01 (0.76-1.33) |  | 130 | 1.02 (0.74-1.41) |  | 67 | 0.98 (0.65-1.45) |  |
|  |  | TT | 55/48 | 1.34 (0.85-2.10) |  | 35 | 1.37 (0.82-2.29) |  | 17 | 1.20 (0.64-2.28) |  |
| *ERCC4* | Rs1800067 | GG | 388/480 | 1.00 | 0.42 | 242 | 1.00 | 0.38 | 132 | 1.00 | 0.76 |
|  |  | AG | 59/70 | 0.90 (0.61-1.32) |  | 37 | 0.88 (0.56-1.37) |  | 20 | 0.93 (0.54-1.60) |  |
|  |  | AA | 2/4 | 0.50 (0.09-2.86) |  | 1 | 0.37 (0.04-3.44) |  | 1 | 0.84 (0.09-7.81) |  |
|  |  | AG/AA | 61/74 | 0.88 (0.60-1.28) | 0.49 | 38 | 0.85 (0.55-1.32) | 0.47 | 21 | 0.92 (0.54-1.57) | 0.77 |
| *LIG3* | Rs1052536 | CC | 205/251 | 1.00 | 0.24 | 121 | 1.00 | 0.60 | 80 | 1.00 | **0.04** |
|  |  | CT | 310/310 | 1.04 (0.80-1.34) |  | 185 | 1.06 (0.78-1.43) |  | 114 | 0.97 (0.68-1.37) |  |
|  |  | TT | 109/133 | 0.79 (0.57-1.10) |  | 72 | 0.88 (0.60-1.29) |  | 30 | 0.56 (0.35-0.91) |  |
| *OGG1* | Rs1052133 | CC | 354/423 | 1.00 | 0.09 | 210 | 1.00 | 0.06 | 129 | 1.00 | 0.44 |
|  |  | CG | 227/229 | 1.23 (0.96-1.57) |  | 145 | 1.33 (1.01-1.76) |  | 77 | 1.12 (0.80-1.57) |  |
|  |  | GG | 33/37 | 1.27 (0.75-2.15) |  | 19 | 1.27 (0.69-2.34) |  | 12 | 1.20 (0.59-2.44) |  |
|  |  | CG/GG | 260/266 | 1.23 (0.98-1.56) | 0.08 | 164 | 1.32 (1.01-1.73) | **0.04** | 89 | 1.13 (0.82-1.56) | 0.45 |
| *NQO1* | Rs1800566 | CC | 425/442 | 1.00 | 0.15 | 255 | 1.00 | 0.33 | 153 | 1.00 | 0.26 |
|  |  | CT | 182/212 | 0.93 (0.72-1.20) |  | 113 | 0.97 (0.73-1.29) |  | 65 | 0.92 (0.65-1.29) |  |
|  |  | TT | 14/32 | 0.54 (0.27-1.06) |  | 9 | 0.56 (0.26-1.25) |  | 5 | 0.53 (0.20-1.43) |  |
| *NOS3* | Rs1799983 | GG | 289/344 | 1.00 | 0.25 | 189 | 1.00 | **0.04** | 93 | 1.00 | 0.67 |
|  |  | GT | 273/264 | 1.08 (0.84-1.38) |  | 154 | 0.93 (0.70-1.24) |  | 106 | 1.31 (0.94-1.83) |  |
|  |  | TT | 57/81 | 0.68 (0.46-1.01) |  | 31 | 0.57 (0.36-0.90) |  | 24 | 0.90 (0.53-1.53) |  |

^b^ OR, odds ratio; 95%CI, 95% confidence interval

^c^ Adjusted for baseline age, race, education, body mass index, family history of prostate cancer, diabetes status, smoking status, physical activity, and alcohol consumption.

^d^ *P-*trend for genetic dose response determined by coding genotypes as having 0, 1, or 2 variant allele, which was subsequently analyzed as an ordinal variable.

^e^ *P* for heterogeneity from dominant or recessive models.
